# The Impact of Nitrile-Specifier Proteins on Indolic Carbinol and Nitrile Formation in Homogenates of *Arabidopsis thaliana*

**DOI:** 10.3390/molecules27228042

**Published:** 2022-11-19

**Authors:** Eleanor C. M. Chroston, Annika Hielscher, Matthias Strieker, Ute Wittstock

**Affiliations:** Institute of Pharmaceutical Biology, Technische Universität Braunschweig, Mendelssohnstr. 1, D-38106 Braunschweig, Germany

**Keywords:** indole glucosinolates, indolic carbinol, indolic nitrile, derivatization, GC-MS, quantification

## Abstract

Glucosinolates, specialized metabolites of the Brassicales including *Brassica* crops and *Arabidopsis thaliana*, have attracted considerable interest as chemical defenses and health-promoting compounds. Their biological activities are mostly due to breakdown products formed upon mixing with co-occurring myrosinases and specifier proteins, which can result in multiple products with differing properties, even from a single glucosinolate. Whereas product profiles of aliphatic glucosinolates have frequently been reported, indole glucosinolate breakdown may result in complex mixtures, the analysis of which challenging. The aim of this study was to assess the breakdown of indole glucosinolates in *A. thaliana* root and rosette homogenates and to test the impact of nitrile-specifier proteins (NSPs) on product profiles. To develop a GC-MS-method for quantification of carbinols and nitriles derived from three prominent indole glucosinolates, we synthesized standards, established derivatization conditions, determined relative response factors and evaluated applicability of the method to plant homogenates. We show that carbinols are more dominant among the detected products in rosette than in root homogenates of wild-type and NSP1- or NSP3-deficient mutants. NSP1 is solely responsible for nitrile formation in rosette homogenates and is the major NSP for indolic nitrile formation in root homogenates, with no contribution from NSP3. These results will contribute to the understanding of the roles of NSPs in plants.

## 1. Introduction

Glucosinolates are plant-specialized metabolites of the order Brassicales, which includes agriculturally and nutritionally important crops of Brassicaceae, such as cabbage, broccoli, kale, mustard and oilseed rape [1,2,3,4]. The presence of glucosinolates in these crops has attracted considerable interest, as they play a role in plant chemical defense against herbivores, and microbial pathogens and are of interest as health-beneficial components in the human diet (e.g., [5,6,7,8,9]). Most of the biological activities associated with glucosinolates are due to their breakdown products, which are formed when glucosinolates are mixed with their hydrolytic enzymes, thioglucoside glucohydrolases known as myrosinases (EC 3.2.1.147) [10,11,12,13]. This can happen in damaged plant tissue or upon reorganization of cellular compartments in intact tissue, where glucosinolate hydrolysis is otherwise prevented by compartmentation or by yet unidentified factors [11,12,14,15]. Owing to their various biological activities, there is considerable interest in detection and quantification of glucosinolate breakdown products in complex biological matrices. Profiles of glucosinolate breakdown products and corresponding analytical methods have been published previously [16,17,18,19]. However, the structural diversity and reactivity of these compounds pose challenges to their analysis.

**Figure 1:**
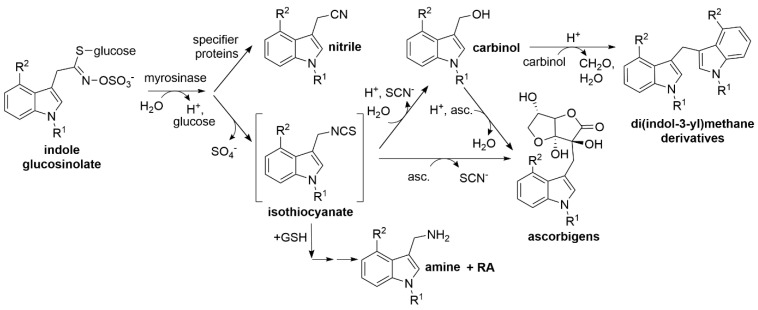
Breakdown of indole glucosinolates. Upon tissue damage or sub-cellular reorganization, indole glucosinolates are hydrolyzed by myrosinases. The aglucones are unstable and rearrange to isothiocyanates which readily react with nucleophiles such as water, ascorbic acid (asc.), and glutathione (GSH) yielding carbinols, ascorbigens, and GSH conjugates, all of which can undergo further reactions. Formation of di(indol-3-yl)methanes or indolic amines and raphanusamic acid (RA) are shown as an example. Ascorbigens can also be formed from carbinols upon reaction with ascorbic acid. Alternatively, the aglucones can be converted to nitriles by specifier proteins. R^1^, R^2^: -H, -OH or -OCH_3_ (see Table 1). The reaction scheme has been adapted from [20] with modifications according to [14].

Major breakdown products of most aliphatic and benzylic glucosinolates are relatively stable isothiocyanates, whereas isothiocyanates derived from indole glucosinolates are unstable and react with various nucleophiles [19,20] (Figure 1). Upon reaction with water, indole carbinols are formed [19,20,21,22]. Many plants possess specifier proteins as additional components of the glucosinolate–myrosinase system [12,23]. These proteins convert glucosinolate aglucones released by myrosinases to nitriles, epithionitriles or organic thiocyanates (jointly referred to as alternative breakdown products), depending on the structure of the aglucone side chain and their substrate/product specificity [24]. As a result, glucosinolate hydrolysis in damaged tissue leads to a mixture of isothiocyanates and derived compounds and nitriles or other specifier protein products [3,18,19,25,26]. The various types of glucosinolate breakdown products differ in their physicochemical properties and biological activities (reviewed in [20,27,28,29]). In order to understand the relevance of glucosinolate breakdown products in plant defense and as components of the human diet, their chemical analytics are an essential prerequisite.

The genetic bases of alternative product formation have mainly been studied in the model plant *Arabidopsis thaliana* L. (Brassicaceae); specifier protein homologs have also been characterized from *Brassica* spec. [12]. Apart from genetic resources, mutant collections and ease of genetic manipulation, *A. thaliana* lends itself as a model to study glucosinolate metabolism owing to its well-characterized glucosinolate–myrosinase system and broad spectrum of glucosinolates and breakdown products [18,30]. *A. thaliana* plants of the Columbia-0 (Col-0) accession produce aliphatic (methionine-derived) and indole (tryptophan-derived) glucosinolates [30]. They possess five genes encoding nitrile-specifier proteins (NSPs), which are differentially expressed in various organs and developmental stages [25,31,32]. Previous GC analysis demonstrated that the major aliphatic glucosinolates (such as 4-(methylsulfinyl)butyl glucosinolate) are mainly converted to isothiocyanates and nitriles in leaf homogenates and mostly to nitriles in homogenates of seeds, seedlings and roots and that the proportion of nitriles depends on functional NSPs [25]. The roots are rich in indole glucosinolates, namely indol-3-ylmethyl glucosinolate (I3M) and its 1-methoxy and 4-methoxy derivatives (1MOI3M and 4MOI3M, respectively) [30,33]. However, their fate in *A. thaliana* homogenates has not been described quantitatively to date due to the lack of appropriate analytical methods. Specifically, most carbinols derived from indole glucosinolates are not sufficiently volatile for direct GC analysis [25,34,35]. Owing to the expression of *NSP1*, *NSP3* and *NSP4* in *A. thaliana* roots [25], we expect that the nitriles indole-3-acetonitrile (I3ACN), 1-methoxyindole-3-acetonitrile (1MOI3ACN) and 4-methoxyindole-3-acetonitrile (4MOI3ACN) are major glucosinolate breakdown products in root homogenates, in addition to the corresponding carbinols indole-3-carbinol (I3C), 1-methoxyindole-3-carbinol (1MOI3C) and 4-methoxyindole-3-carbinol (4MOI3C), as well as other isothiocyanate-derived compounds.

The aim of this study was to assess breakdown product formation from the three major indole glucosinolates of *A. thaliana* roots and rosettes in order to investigate the impact of *NSP* genes on indolic breakdown product profiles under the conditions previously used to establish the fate of aliphatic glucosinolates in *A. thaliana* homogenates [25]. In a previous in vitro study, I3M hydrolysis yielded 70–80 mol-% I3C at pH 6, depending on the presence or absence of ascorbic acid, followed by approximately 25 mol-% ascorbigen in the presence of ascorbic acid [19]. These results, together with NSP expression in the studied organs, led us to expect nitriles and carbinols as major products. As a first step, we developed a method for quantitative analysis of the carbinols and nitriles derived from I3M, 1MOI3M and 4MOI3M (Figure 1 and Table 1) by GC-MS. Using synthetic standards of the six analytes of interest, we tested and optimized derivatization conditions, compared the analytical responses based on reconstructed ion chromatograms (RIC) and evaluated the applicability of the method to plant extracts using *A. thaliana* wild-type plants and a mutant line deficient in indole glucosinolate biosynthesis. Next, we applied the method to *A. thaliana* mutant lines previously shown to be deficient in nitrile formation from major aliphatic glucosinolates [25] to test whether these plants are also compromised in nitrile formation from indole glucosinolates.

## 2. Materials and Methods

### 2.1. Chemicals

Indoline (99%), I3C (97%) and I3ACN (97%) were purchased from VWR International (Darmstadt, Germany), 4-methoxyindole (95%) from abcr GmbH (Karlsruhe, Germany), indole-3-carbonitrile (I3CN; 98%) from Alfa Aesar-Thermo Fisher Scientific (Dreieich, Germany) and benzonitrile (≥99%) from Sigma Aldrich (Taufkirchen, Germany). *N*-Methyl-*N*-(trimethylsilyl)trifluoroacetamide (MSTFA) was purchased from Alfa Aesar-Thermo Fisher Scientific (Dreieich, Germany), and 1% trimethylchlorosilane (TMCS) in MSTFA was purchased from Sigma Aldrich (Taufkirchen, Germany). 4-Hydroxybenzyl glucosinolate (99%) was purified from seeds of *Sinapis alba* L. (Brassicaceae), and its identity was confirmed by HPLC-DAD in comparison with an authentic standard.

### 2.2. Plant Material

*A. thaliana* (L.) HEYN. plants of the Col-0 accession (wild-type), as well as mutant plant lines *cyp79b2 cyp79b3* [36], *cyp79b2 cyp79b3 myb28 myb29* [37,38], *nsp1-1* and *nsp3-2* [25], were grown with a 10 h photoperiod (100 µmol m^−2^ s^−1^ photosynthetically active radiation) at 22 °C and 60–70% relative humidity in a controlled environment chamber (Percival AR-66L). All plants were sown onto a mixture of germination potting compost (Compo, Münster, Germany; 80%), sand (10%) and vermiculite (10%) supplemented with 2 g/L Triabon (Compo) and 1 g/L Sierrablen (Scotts, Heerlen, Netherlands) fertilizer. Three weeks after sowing, the plants were transferred to pots (5 cm diameter, one plant per pot) filled with Seramis clay granules (Seramis, Mogendorf, Germany) soaked in Hoagland’s solution and grown for an additional three weeks [39,40].

Six weeks after sowing, plants were harvested by submerging the complete content of the pot in Hoagland’s solution. Remaining clay granules were carefully plucked out of the root cluster with tweezers. Roots and rosettes were separated, weighted and used directly for subsequent analysis of glucosinolate breakdown products or freeze-dried for analysis of glucosinolates. Rosettes were analyzed individually unless otherwise stated, whereas roots were pooled from six plants to afford one sample.

### 2.3. Chemical Synthesis of Indolic Standard Compounds

Compounds **3** (92%), **4** (96%), **5** (95%) and **6** (97%) were synthesized according to [41,42,43] as described below. Intermediates and products were purified by flash column chromatography on silica gel using dichloromethane as eluent. The identity of the synthesized standards was confirmed by NMR spectroscopy (Avance III HD 500 and Avance III 400, Bruker Corporation, Billerica, MA, USA) and MS (3200 QTRAP LC-MS/MS system, Applied Biosystems MDS Sciex, Darmstadt, Germany) (Supplementary Material Data S1). Purity was evaluated by HPLC on an Agilent HP1200 Series instrument equipped with a Chromolith RP-18e column (100 × 3 mm, particle size 5 µm; Merck) with a 5–3 guard cartridge and coupled to a DAD detector. Elution was accomplished with a water–acetonitrile gradient (Supplementary Material Table S1). Peak areas were determined at 254.8 nm for optimal representation of impurities.

1-Methoxyindole was synthesized from indoline according to [41] and converted to 1MOI3C (**3**) via a 3-formyl intermediate, followed by NaBH_4_-mediated reduction according to [42] (Supplementary Material Scheme S1). Alternatively, a 3-acetonitrile moiety was introduced via 1-methoxygramine as intermediate according to [43] to yield 1MOI3ACN (**4**) (Supplementary Material Scheme S2).

4-Methoxyindole was formylated at position 3, followed by NaBH_4_-mediated reduction based on [42] to 4MOI3C (**5**) (Supplementary Material Scheme S3). Alternatively, 4-methoxyindole was converted to its 3-acetonitrile derivative via 4-methoxygramine as an intermediate based on [43] to yield 4MOI3ACN (**6**) (Supplementary Material Scheme S4).

### 2.4. Derivatization of Standard Compounds

Standard compounds were dissolved in methanol. An appropriate aliquot of a mixture of all seven compounds was dried in an air stream at room temperature and dissolved in 75 µL derivatization reagent (MSTFA, MSTFA/pyridine 2 + 1, or 1% TMCS in MSTFA). Samples were incubated at room temperature or 70–85 °C for varying durations and analyzed by GC-MS and/or GC-FID.

### 2.5. GC-MS and GC-FID

Derivatized samples were analyzed by GC-MS on an Agilent Technologies 6890N (Agilent Technologies Deutschland GmbH, Böblingen, Germany) gas chromatograph equipped with a ZB-5MS capillary column (30 m × 0.25 mm; ft 0.25 µm; Phenomenex, Aschaffenburg, Germany) coupled with an Agilent Technologies 5975B mass spectrometer. After injection at 270 °C (injection volume, 1 µL; split ratio, 1:5), separation was achieved with the following temperature program: 70 °C for 3 min, 10 °C min^−1^ to 310 °C and a 3 min final hold. Helium was used as a carrier gas. EI-MS was performed with the following settings: ionization voltage of 70 eV, ion source temperature of 230 °C, interface temperature of 290 °C and EI full scan (range *m/z* 41.0–600.0). The compounds were identified by retention times and an in-house mass spectra library of derivatized authentic or synthesized standard compounds (Supplementary Material Data S2). Quantification was based on the RIC of the molecular ions. GC-FID (only applied during establishment of the derivatization conditions) was conducted on an Agilent Technologies 6890N gas chromatograph using the same column and temperature program with splitless injection. Helium was used as a carrier gas, and the detector was operated at 300 °C. As the analytes coeluted with other compounds in plant homogenates, RIC peak areas from GC-MS runs had to be used for quantification instead of FID peak areas to ensure specificity upon analysis of plant homogenates.

### 2.6. Determination of Response Factors

Response factors were derived from calibration lines of each compound of interest and the internal standard I3CN. For each of two independent experiments, all compounds were weighed, dissolved to 100 mM in methanol and stored at −20 °C. For each dilution series, a 10-fold and a 100-fold dilution of each stock was prepared, and aliquots were combined to prepare standard mixtures (one from the 10-fold and one from the 100-fold dilution). An appropriate volume of the relevant mixture was brought to dryness to obtain samples composed of 1, 10, 25, 50 or 100 nmol of each compound. After addition of 75 µL MSTFA/pyridine (2 + 1), samples were incubated for 1 h at 70 °C and analyzed by GC-MS. Occasionally, this revealed incomplete derivatization (likely due to the presence of water in the samples). In such cases, samples were reanalyzed after an additional incubation at 85 °C. Peak areas determined from the RIC of the molecular ions were plotted against the molar amounts. The slope of the regression line was used to calculate the response factor as the slope ratio (internal standard/analyte). In experiment 1, measurements were performed with four dilution series, and data were pooled to generate one regression line per compound. In experiment 2, three dilution series were analyzed with three replicates each, and data for replicates were pooled to obtain one regression line per dilution series and compound.

### 2.7. Analysis of Indole Glucosinolate Breakdown Products in Root and Leaf Homogenates

Fresh plant material was ground with sea sand (p.a.; Merck, Darmstadt, Germany) using a mortar and pestle in 50 mM MES buffer, pH 6.0 (100 µL buffer per 100 mg plant material). I3CN (25 or 20 µL of 1 nmol µL^−1^ in MeOH) and benzonitrile (30 or 25 µL of 100 ng µL^−1^ in MeOH) were added as internal standards. After 10 min incubation at room temperature, plant parts were removed by centrifugation, and the aqueous solution was extracted twice with 750 µL dichloromethane. Organic phases were passed over Na_2_SO_4_, dried in an air stream to complete dryness, dissolved in 75 µL MSTFA/pyridine (2 + 1), incubated at 70 °C for 1 h and analyzed by GC-MS. Indolic breakdown products were identified by comparison of mass spectra and retention times with those of the standard mixture. Quantification was achieved based on peak areas determined from the RIC of the molecular ions using the experimentally determined response factors (Supplementary Material Table S2).

### 2.8. Generation of Di(indol-3-yl)methane Derivatives

A mixture of I3C (**1**), 1MOI3C (**3**) and 4MOI3C (**5**) (100 nmol of each compound) was dissolved in 500 µL 50 mM MES buffer, pH 5, and left at 22 °C for 17 h. The mixture was then extracted twice with dichloromethane, and the pooled organic phases were subjected to derivatization as described above for plant homogenates. The preparation was analyzed by GC-MS under the conditions described above (Supplementary Material Table S3). Retention times and mass spectra were used to identify di(indol-3-yl)methane derivatives in plant homogenates.

### 2.9. Analysis of Glucosinolates

Glucosinolate profiles were determined as described previously [25]. Briefly, desulfoglucosinolates were prepared from extracts of freeze-dried plant material (three rosettes or roots of six plants were pooled, resulting in one sample per genotype, organ and experiment) and analyzed by HPLC on an Agilent HP1200 Series instrument equipped with a LiChrospher RP18 column (250 mm × 4.6 mm, 5 µm particle size; Wicom, Heppenheim, Germany) and coupled with a DAD detector. Elution was accomplished with gradient 1 (Supplementary Material Table S4) and gradient 2 (for complete separation of desulfo-5-methylsulfinylpentyl glucosinolate and desulfo-4MOI3M; Supplementary Material Table S5). Quantification was based on the peak area at 229 nm relative to that of an internal standard (4-hydroxybenzyl glucosinolate) using response factors 0.25 for indole glucosinolates (as recommended in [44], p. 55) and 0.50 for 4-hydroxybenzyl glucosinolate based on experimental studies reviewed in [45].

### 2.10. Statistics

Statistical analyses were performed with OriginPro 2021 (OriginLab Corporation, Northampton, MA, USA). Homogeneity of variance was assessed using ANOVA with the Brown–Forsythe Test. Significant differences in pairwise comparisons between wild-type and mutant were identified using a two-sample *t*-test (in the case of normal distribution) and the Mann–Whitney test (when normal distribution was not confirmed).

## 3. Results

### 3.1. Optimized Derivatization Makes Indolic Carbinols and Nitriles Accessible for GC

**Figure 2:**
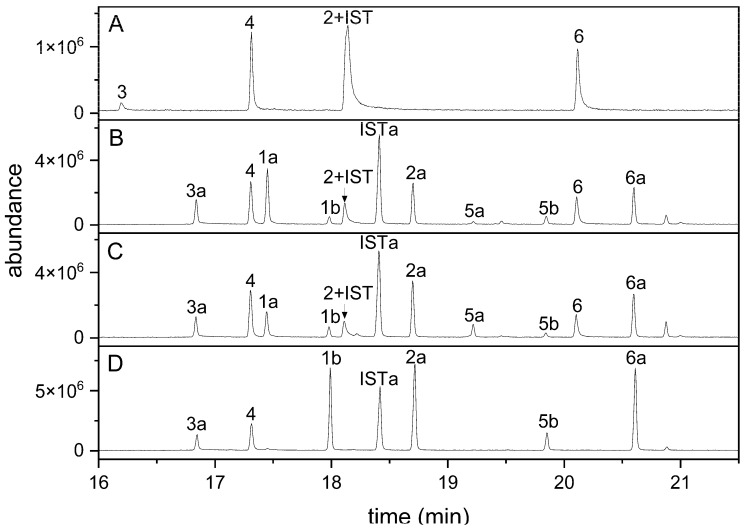
GC detection of indolic carbinols and nitriles after derivatization with different reagents. A mixture of the six indolic glucosinolate breakdown products and I3CN (internal standard, IST) was analyzed by GC-MS without derivatization (**A**) and after incubation in MSTFA (**B**), MSTFA + 1% TMCS (**C**) or MSTFA/pyridine (2 + 1) (**D**) at 70 °C for 1 h. GC-MS total ion current (TIC) traces are shown. Compound numbers the same as in Table 1; suffix a indicates silylation in one position, and suffix b indicates silylation in two positions. Carbinols 1 and 5 can carry two TMS groups; the other compounds can be silylated once, with the exception of 4 (no silylation).

When indolic carbinols and nitriles (**1**–**6**) including the internal standard I3CN were analyzed by GC-MS, all four nitriles were detectable (Figure 2A). In contrast, only one of the three carbinols was detected, presumably owing to their relatively high polarity (Figure 2A). To reduce the polarity of these compounds and to make them suitable for GC analysis, we generated their silylated derivatives using MSTFA and analyzed a mixture of all seven compounds. Carbinols without substitution in position 1 (**1**, **5**) can carry two TMS groups (or only one of the two when incompletely derivatized), whereas the corresponding nitriles (**2**, **6**) can only be silylated once. 1MOI3ACN (**4**), in which position 1 is occupied, does not provide a target for silylation. Based on the molecular ions obtained upon GC-MS, the use of just MSTFA as derivatization reagent at 70 °C for 1 h resulted in complete silylation of **3** but incomplete silylation of **1**, **2**, **5**, **6** and the internal standard, as indicated by the presence of several peaks for each of these educts (representing educt and silylated derivatives; Figure 2B). Compounds **1** and **5** appeared to be silylated preferentially at one of the two possible positions (-NH, -CH_2_OH), as we only detected one monosilylated and the disilylated product. As alcoholic groups are more easily silylated than amines, we assume that peaks **1a** and **5a** (Figure 2B) represent derivatives with silylated carbinol function [46]. We therefore tested whether longer reaction times and/or the presence of catalysts would improve the conversion. An elongated reaction time did not yield satisfying results (data not shown). When MSTFA was supplemented with 1% TMCS and compounds were derivatized at room temperature or at 70 °C for up to 4 h, no improvement was achieved (Figure 2C). However, the use of MSTFA/pyridine (2 + 1) at 70 °C for at least 1 h resulted in a chromatogram with only seven peaks as expected for complete derivatization (Figure 2D). Accordingly, peak areas of the fully silylated derivatives of **1**, **2**, **5** and **6** were larger than those obtained after derivatization with MSTFA or MSTFA/TMCS. Derivatization with MSTFA/pyridine (2 + 1) at room temperature or for only 30 min at 70 °C did not result in complete silylation (data not shown). Incubations in MSTFA/pyridine (2 + 1) at 70 °C for up to 3 h led to slightly increased peak areas compared to those obtained with a 1 h reaction time but no change relative to the peak area of the internal standard (Supplementary Material Figure S1). A higher reaction temperature (85 °C) did not increase the reaction rate or yield. Therefore, we decided to use MSTFA/pyridine (2 + 1) at 70 °C for 1 h in all further experiments.

### 3.2. Quantitative Analysis of Indolic Carbinol and Nitrile Standards

To enable absolute quantification of indolic glucosinolate breakdown products in plant homogenates, we conducted calibrations with the standard compounds using RIC peak areas to ensure specificity despite the complex matrix. As most of the relevant indolic standard compounds are not generally available for repeated calibrations and to account for the complex matrix of the plant homogenates, we decided to base quantification on an internal standard and therefore determined response factors of our analytes relative to the internal standard I3CN. Mixtures of 1, 10, 25, 50 or 100 nmol of each compound were subjected to derivatization followed by GC-MS analysis. All compounds were detected as single peaks (representing the completely silylated derivative) at all tested concentrations, and there was a linear relation between the used amount and peak area obtained up to at least 50 nmol substance in the sample subjected to derivatization. We used the slopes of the calibration lines to calculate response factors of analytes **1**–**6** relative to I3CN. In the case of **1**, **2**, **5** and **6**, this worked well and showed similar responses for the compounds and the internal standard (corresponding to response factors of approximately 1), despite some variation between replicates and independent calibrations (Supplementary Material Table S2). In the case of **3** (1MOI3C) and **4** (1MOI3ACN), independent calibrations and repeated measurements showed lower slopes for these compounds than for the internal standard. The extent of this difference varied considerably (Supplementary Material Table S2), probably due to the hygroscopicity of the synthesized substances and the difficulties associated with the limited amounts of available substance. Based on our results, we propose consideration of approximate response factors of 2.5 and 2 (relative to I3CN), respectively, to roughly estimate absolute amounts of 1MOI3C and 1MOI3ACN.

### 3.3. The Method Is Applicable to Plant Homogenates

**Figure 3:**
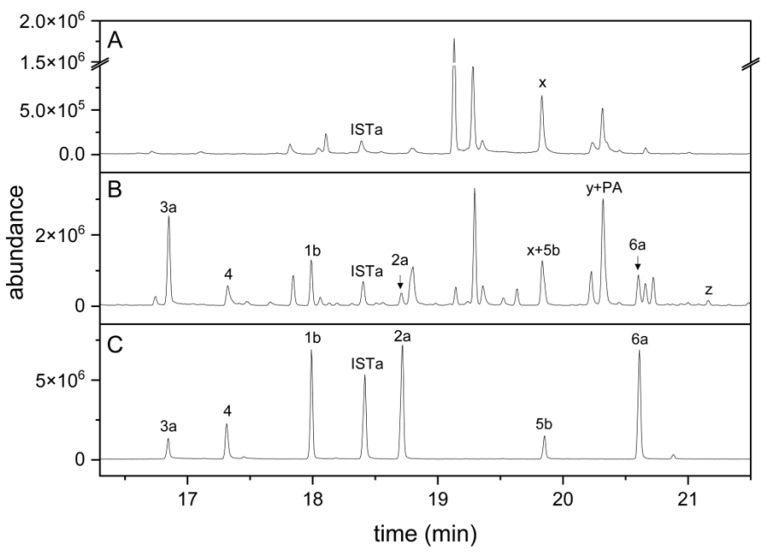
Indole glucosinolate breakdown products in root homogenates of *A. thaliana cyp79b2 cyp79b3* and Col-0 plants. (**A**) *cyp79b2 cyp79b3*; (**B**) Col-0; (**C**) mixture of synthetic standards. Dichloromethane extracts of root homogenates or the standard mixture were dried, derivatized and analyzed by GC-MS. TIC traces are shown. Numbers refer to compounds in Table 1 (detected after complete silylation), and IST refers to the I3CN internal standard. Suffix a indicates silylation in one position, and suffix b silylation in two positions. x, unidentified compound. Peak y represents threefold silylated 4OHI3C plus silylated palmitic acid (PA), and peak z represents twofold silylated 4OHI3ACN.

To test whether the established method can be used to detect indolic carbinols and nitriles in plant homogenates, we homogenized roots of aeroponically grown *A. thaliana* Col-0 wild-type and *cyp79b2 cyp79b3* mutant plants and subjected dichloromethane extracts of the homogenates to the derivatization procedure. In samples from Col-0 roots, six peaks corresponding to the carbinols and nitriles derived from I3M, 1MOI3M and 4MOI3M were detected (Figure 3B) based on the mass spectra and retention times in comparison to those of the standard compounds (Figure 3C). In addition, we found peaks that we tentatively assigned to the carbinol (4OHI3C) and nitrile (4OHI3ACN) of 4-hydroxyindol-3-yl-methyl glucosinolate (4OHI3M), a minor root glucosinolate, in silylated form (Figure 3B), based on the mass spectra and comparison with root homogenates spiked with 4OHI3M (data not shown). Di(indol-3-yl)methane derivatives (Supplementary Material Table S3) were only detected in traces (see below). None of the peaks derived from indolic breakdown products was present in samples from *cyp79b2 cyp79b3* plants, which are defective in indole glucosinolate biosynthesis (Figure 3A). All plant samples contained a compound (“x” in Figure 3A,B) with the same retention time as **5b** but with a different mass spectrum. In case of wild-type samples, this compound co-eluted with **5b**. In samples from *cyp79b2* and *cyp79b3* plants, the corresponding peak represented compound x. Compound x was not present in derivatized samples from roots of *cyp79b2 cyp79b3 myb28 myb29* plants defective in both aliphatic and indole glucosinolate biosynthesis (data not shown), indicating its possible origin from aliphatic glucosinolates. Spiking experiments with *cyp79b2 cyp79b3* root homogenates showed that the synthetic standards, except for 1MOI3ACN, were detectable at <3 pmol per split injection (corresponding to 1 nmol per 75 µL sample), despite the plant matrix. In the case of 1MOI3ACN, the detection limit was about fivefold higher (15 pmol, corresponding to 5 nmol per 75 µL sample). Taken together, the six indolic carbinols and nitriles of interest, as well as the internal standard, can be detected in root homogenates by GC-MS after derivatization according to the above method. In addition, the carbinol and nitrile of the minor glucosinolate, 4OHI3M, are detected. Owing to the lack of standards for these compounds, they were not included in quantitative analyses.

We conducted quantitative analyses using the above method including the determined response factors (Supplementary Material Table S2) to evaluate recovery of the analytes from root and leaf homogenates. Specifically, we compared the levels of indole glucosinolates quantified by HPLC after desulfation with the total amount of carbinols and nitriles detected in homogenates using the above method. The absolute quantities varied considerably between experiments, but on average, the levels of detected products (carbinol + nitrile, in nmol/g fw) corresponded approximately to the content of the respective glucosinolates in roots and rosettes of wild-type and *nsp3-2* plants (Supplementary Material Figures S2 and S3). Indole glucosinolate content in wild-type plants was in the expected range based on the literature [30,33]. However, in *nsp1-1* root homogenates, we detected only about half of the I3M and 4MOI3M breakdown product amounts expected based on glucosinolate content (Supplementary Material Figure S2B,C; see also below), together with traces of di(indol-3-yl)methane derivatives (see below). This indicates low recovery of the corresponding carbinols and/or formation of additional products, which remained unidentified in the present study. As such a difference was not observed in rosettes (Supplementary Material Figure S3B,C), formation of additional products upon I3M and 4MOI3M hydrolysis in root homogenates is a likely scenario. Di(indol-3-yl)methane derivatives were only present in traces or not detected in both root and rosette homogenates of all studied genotypes (Supplementary Material Table S6). They coeluted with other (dominating) substances not related to indole glucosinolates based on the mass spectra (Supplementary Material Figure S4).

### 3.4. Indole Glucosinolate Substitution Patterns and NSP Deficiency Affect Indolic Carbinol/Nitrile Proportion in A. thaliana Root Homogenates

**Figure 4:**
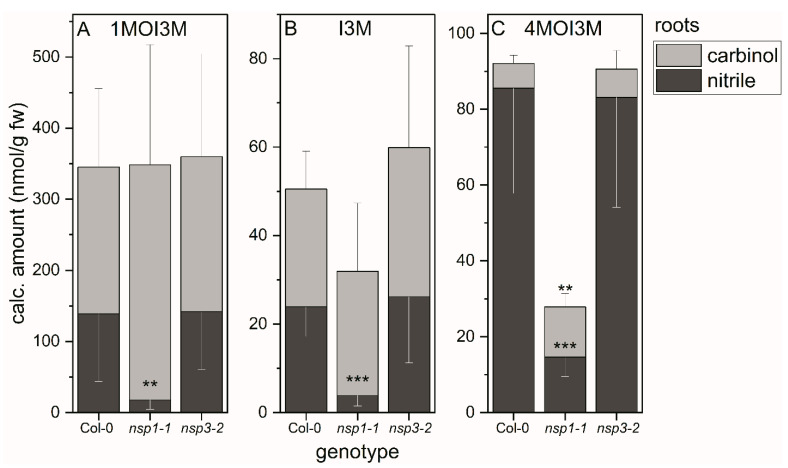
Breakdown of indole glucosinolates in homogenates of roots of WT, as well as *nsp1-1* and *nsp3-2* mutant plants. Absolute amounts of carbinols and nitriles derived from indole glucosinolates 1MOI3M (**A**), I3M (**B**) and 4MOI3M (**C**) relative to root fresh weight. Means ± SD of *N* = 6 independent experiments (one sample composed of roots from six plants per experiment). Significant differences between wild-type and mutants are indicated separately for each carbinol and nitrile by asterisks (**, *p* <0.01; ***, *p* < 0.001; two-sample *t*-test; Mann–Whitney test when normal distribution could not be confirmed).

We applied the above method to evaluate whether the substitution patterns of indole glucosinolates affect the proportion of the two product types, carbinols and nitriles, in *A. thaliana* wild-type root homogenates. In the case of 1MOI3M and I3M, the corresponding nitriles accounted for 40–50% of the detected breakdown products (Figure 4A,B). In the case of 4MOI3M, about 90% of the detected breakdown products were the nitrile (Figure 4C). As comparison of total amount of detected breakdown products with glucosinolate content had indicated complete recovery of breakdown products in wild-type root homogenates (see above and Supplementary Material Figure S2), this indicates that the proportion of nitrile formed in root homogenates depends on the substitution pattern of the indole glucosinolate. Next, we investigated whether deficiency in functional NSP1 or NSP3 affects nitrile formation from indole glucosinolates in root homogenates. In homogenates of *nsp1-1* roots, the amounts of 1MOI3ACN, I3ACN and 4MOI3ACN were reduced by 80–90% compared to root homogenates of the wild-type (*p* < 0.001) (Figure 4). In contrast, the indolic nitrile levels in *nsp3-2* root homogenates were unchanged relative to the wild-type (Figure 4). Thus, NSP1 contributes most of the nitrile-forming activity upon breakdown of 1MOI3M, I3M and 4MOI3M in *A. thaliana* root homogenates, whereas NSP3 does not play a major role under the tested conditions. A small reduction in nitrile formation in *nsp3-2* may have remained undetected due to NSP1 activity. As *nsp1-1* is a knockout mutant with no *NSP1* expression [25,32], either NSP3 and/or another NSP, for example NSP4, which is also root-expressed, is likely responsible for formation of the small amounts of nitriles in *nsp1-1* root homogenates. However, NSP-independent nitrile formation by spontaneous rearrangement of the aglucone cannot be ruled out.

If NSPs are non-functional, we expect glucosinolate breakdown to yield isothiocyanates and derived products such as carbinols instead of nitriles. Accordingly, we found increased levels of 1MOI3C in *nsp1-1* root homogenates compared to the wild-type, although the increase was not significant (Figure 4A). The larger amount of 1MOI3C made up for the lack of the corresponding nitrile in terms of the total amount of detected 1MOI3M breakdown products (carbinol + nitrile) relative to the wild-type. In the case of I3M and 4MOI3M, the amount of detected carbinol did not increase or not to the same extent as the nitrile amount decreased (Figure 4B,C), in agreement with our finding that amounts of I3M- and 4MOI3M-derived carbinols and nitriles in *nsp1-1* root homogenates were much lower than expected based on the content of these glucosinolates in the starting material (see above, Supplementary Material Figure S2). This indicates the formation of products not captured by this method, either from the glucosinolate aglucones or upon further metabolism of the isothiocyanates or I3C and 4MOI3C, respectively, in *nsp1-1* root homogenates. Under the tested conditions, di(indol-3-yl)methane derivatives are not formed as major products, even in root homogenates of NSP-deficient *A. thaliana* (Supplementary Material Table S6).

### 3.5. Methyl Ethers of Carbinols Are Formed in Root Homogenates and in an Aqueous Carbinol Standard Mixture

Upon evaluation of the chromatograms of derivatized root homogenates of wild-type plants, we detected additional peaks that were assigned to derivatized methyl ethers of 1MOI3C, I3C and 4MOI3C based on their mass spectra (Supplementary Material Figure S5). Owing to the lack of standards, we were not able to quantify these compounds. Based on the peak areas (TIC, Supplementary Material Figure S5) in comparison to those of their (non-methylated) precursor compounds, we estimate that a proportion of 5–15% of 1MOI3C (**3**) and I3C (**1**) undergoes methylation. In the case of 4MOI3C-methylation, such an estimation is impossible, as 4MOI3C (**5**) coelutes with another substance (x in Figure 3 and Supplementary Material Figure S5), and its quantity is therefore not well-represented by the TIC peak area. Although not present in derivatized samples of methanolic standard mixtures, the tentatively identified methyl ethers of 1MOI3C, I3C and 4MOI3C were also detected in considerable amounts when a mixture of the carbinols was left in MES buffer, pH 5.0, for 17 h and subjected to GC-MS after derivatization.

### 3.6. NSP1 Is Solely Responsible for Indolic Nitrile Formation in Rosette Homogenates of A. thaliana

**Figure 5:**
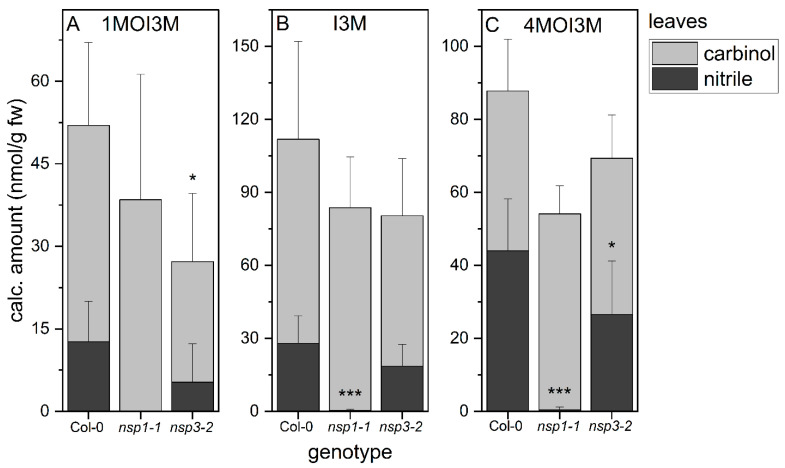
Breakdown of indole glucosinolates in rosette homogenates of wild-type and *nsp1-1* and *nsp3-2* mutant plants. Absolute amount of carbinols and nitriles derived from indole glucosinolates 1MOI3M (**A**), I3M (**B**) and 4MOI3M (**C**) relative to fresh weight. Results of three independent experiments with three plants per experiment. Means ± SD (*N* = 9). Significant differences between the wild-type and mutants are indicated separately for each carbinol and nitrile by asterisks (*, *p* < 0.05; ***, *p* < 0.001; two-sample *t*-test; Mann–Whitney test when normal distribution could not be confirmed).

As previous analyses of glucosinolate breakdown products in rosette homogenates were limited to aliphatic glucosinolates, we now applied our method for indolic carbinol and nitrile quantification to rosette homogenates. In wild-type rosette homogenates, carbinols accounted for about 75% of the detected indolic breakdown products of 1MOI3M and I3M and for about 50% of the detected products of 4MOI3M (Figure 5). Thus, the substitution pattern of the parent glucosinolate affected the product proportion to some extent. In homogenates of *nsp1-1* rosettes, we detected almost exclusively the carbinols of all three indole glucosinolates, in addition to trace amounts of the corresponding nitriles (Figure 5). This suggests that NSP1 is responsible for indolic nitrile formation in rosette homogenates, with no contribution from other NSPs. Rosette homogenates of *nsp3-2* plants had, on average, lower levels of indolic nitriles than homogenates of wild-type rosettes, although this was only significant in the case of 4MOI3ACN (*p* < 0.05), largely in agreement with the lack of nitriles in *nsp1-1* rosette homogenates (Figure 5). As nitrile formation is abolished in *nsp1-1* rosettes, we expected to find larger amounts of the carbinols in *nsp1-1* homogenates than in wild-type homogenates. The levels of carbinols derived from 1MOI3M, I3M and 4MOI3M in *nsp1-1* homogenates were, on average, equal to or slightly higher than those in wild-type homogenates, but in no case were the amounts increased to the same extent as the nitrile amount decreased (Figure 5). Di(indol-3-ylmethyl)methane derivatives were only found in traces in both wild-type and mutant rosette homogenates (Supplementary Material Table S6). These results suggest the formation of additional metabolites, e.g., as a result of further reactions of the carbinols or side reactions initiated by the reactivity of their isothiocyanate precursors (Figure 1).

## 4. Discussion

Breakdown of indolic glucosinolates upon tissue damage and in undamaged tissue has been the subject of a considerable amount of research (e.g., [14,17,19,20,22,47]). The reactivity of indolic isothiocyanates results in multiple possible metabolic routes that need to be considered comprehensively to understand their interconnection, regulation, biological roles and possible impacts on plant defense and human health [20,28,29]. In the present work, we focused on a small set of products of indole glucosinolate breakdown, namely nitriles and carbinols. Whereas indolic nitriles are detected well by GC methods, the corresponding carbinols are often inaccessible to GC, which has prevented comparative analysis of indolic breakdown products, for example, in homogenates of wild-type and mutant *A. thaliana* lines, in which these compounds are expected as major breakdown products of indole glucosinolates, as well as in *Brassica* species [25,34,35]. Enzyme assays with thioglucosidases and indole glucosinolates often rely on determination of substrate decay but forego product detection, owing to the difficulty of detecting and quantifying the various possible products [14]. An HPLC method has been used to detect I3C and I3ACN as products of enzymatic reactions [17] but poses specificity problems when applied to plant extracts. In the present study, we applied chemical derivatization to make indolic carbinols accessible to GC analysis. After optimization, derivatization of synthetic carbinols and nitriles of the major *A. thaliana* indole glucosinolates yielded their fully silylated products. Quantification was achieved by calibration based on GC-MS RIC peak areas and determination of response factors relative to an internal standard. We used the *cyp79b2 cyp79b3* mutant of *A. thaliana* deficient in indolic glucosinolate biosynthesis to confirm the applicability of the method to plant homogenates spiked with synthetic standards (Figure 3). Synthetic standards were detectable at amounts even below 3 pmol per split injection, with the exception of 1MOI3ACN, with a detection limit of 15 pmol. For *A. thaliana* wild-type roots and rosettes, we found that the total amount of indolic carbinols and nitriles in homogenates corresponded well with the content of the precursor glucosinolates (Supplementary Material Figures S2 and S3), in agreement with a high level of analytical recovery and a high proportion of these breakdown products relative to other possible products not captured by the method. Di(indol-3-yl)methane derivatives were only detected in traces under our experimental conditions (Supplementary Material Figure S4, Supplementary Material Table S6), while our experiments were not set up to analyze the formation of ascorbigens. We can therefore not exclude the possibility that considerable amounts of ascorbigens might also be formed in *A. thaliana* root and leaf homogenates. We detected low levels of the methyl ethers of 1MOI3C, I3C and 4MOI3C in homogenates of wild-type roots (Supplementary Material Figure S5) and leaves (data not shown). Methyl ethers were also formed when root homogenates of *cyp79b2 cyp79b3* plants were spiked with the carbinols and when an aqueous mixture of the carbinol standards was derivatized after 17 h incubation at pH 5.0. It remains to be investigated whether formation of methyl ethers is solely the result of non-enzymatic reactions, likely during derivatization, or whether methyltransferase activity in the plant homogenate contributes to the formation of these compounds. We also detected the nitrile and carbinol of the minor *A. thaliana* root glucosinolate 4OHI3M but were not able to quantify these products due to the lack of an authentic standard.

The proportion of nitriles and carbinols in homogenates differed between *A. thaliana* roots and rosettes and between products of different indole glucosinolates (Figure 4 and Figure 5). For all three major indole glucosinolates, the proportion of nitrile was higher in wild-type root homogenates than in wild-type rosette homogenates, in agreement with the results reported for the major aliphatic glucosinolates in roots and rosettes [25]. Among the three indole glucosinolates, the highest proportion of nitrile was detected with 4MOI3M in both root and rosette homogenates (on average, 95 and 55%, respectively). For 1MOI3M and I3M, we identified carbinols as the major products (about 75% of detected products) in rosette homogenates, whereas carbinols and nitriles contributed equally to the total breakdown product amount in root homogenates. The high proportion of nitriles formed from 4MOI3M indicates that the NSPs might possess some specificity and might convert the aglucone of 4MOI3M with higher efficiency than those of 1MOI3M and I3M. In vitro experiments with purified recombinant NSPs are needed to test this hypothesis. As an alternative explanation, the apparent “preference” of nitrile formation from 4MOI3M might depend on the substrate specificity of the involved thioglucosidase(s) and their ability to interact with the NSPs. Therefore, future work should also consider the ‘classical’ root myrosinases TGG4 and TGG5, as well as ‘atypical’ myrosinases, such as BGLU23 (PYK10), BGLU26 (PEN2) and their homologs, in biochemical investigations on product formation and protein–protein interactions. A “preference” for nitrile formation in the case of 4MOI3M but not 1MOI3M and I3M could also be due to spatial organization of the glucosinolate–myrosinase system at the tissue level, that is, accumulation of only 4MOI3M in vicinity of a certain combination of a myrosinase and an NSP. Differential accumulation of 4MOI3M, together with specific myrosinase(s) and certain NSPs, could lead to specific pathways of product formation in planta. However, we observed this “preference” in homogenates of entire roots and rosettes and therefore assume that biochemical properties of the enzymes or their interaction rather than compartmentation play at least a major role.

The fact that the nitrile proportion is generally higher in *A. thaliana* root homogenates than in rosette homogenates raises questions about the possible roles of nitrile formation in roots. With root homogenates, we basically imitated tissue damage as a trigger for glucosinolate breakdown, similar to the situation that might occur when a herbivore feeds on roots. As previous work has demonstrated glucosinolate breakdown in undamaged tissue, future experiments should address glucosinolate breakdown pathways in intact roots and evaluate the biological roles of the formed products. Glucosinolates and/or their breakdown products might be involved in beneficial interactions with soil organisms, which would likely require exudation of glucosinolates and/or glucosinolate breakdown in intact tissue and exudation of the products. Nitriles have repeatedly been reported to be present in medium of hydroponic cultures, supporting such roles [48]. A recent study revealed positive and negative correlations of the content of I3C, as well as of a number of intact glucosinolates in root extracts, with indicator operational taxonomic units in the rhizosphere microbiome of two accessions of *A. thaliana* [49].

Our analyses revealed that NSP1 is solely responsible for nitrile formation from the three indole glucosinolates under consideration, 1MOI3M, I3M and 4MOI3M, in *A. thaliana* rosette homogenates (Figure 5). The corresponding nitriles were not or only barely detectable in *nsp1-1* rosette homogenates. In contrast, indolic nitriles were strongly reduced but still detectable in root homogenates of *nsp1-1* plants (Figure 4), in agreement with the previous finding that rosettes express only *NSP1*, whereas roots express *NSP1*, *NSP3* and *NSP4* [25]. Breakdown product formation in *nsp3-2* root homogenates did not differ from that in the wild-type. Therefore, NSP4 (rather than NSP3) is likely to contribute to nitrile formation from indole glucosinolates in root homogenates. To test this hypothesis, a knockout mutant with no *NSP4* expression would be helpful, but such a mutant is presently not available.

The observation that the amounts of carbinols formed in *nsp1-1* root homogenates do not increase significantly and/or to the same extent as nitriles decrease indicates that products not captured by our method might be formed, especially when nitrile formation is reduced. These products could be derived from the indole glucosinolate aglucones, the corresponding isothiocyanates or the carbinols [19,22,28]. As the breakdown products were well-recovered relative to the indole glucosinolate levels in wild-type roots and rosettes, irrespective of the range of nitrile–carbinol proportions in homogenates (but not in *nsp1-1* roots), our results point at a metabolic link between deficiency in nitrile formation in roots and formation of such products. A possible scenario includes accumulation of indolic isothiocyanates, their reaction with glutathion, cleavage of the conjugate and formation of the corresponding indolic amine and raphanusamic acid (Figure 1) [14,50]. Raphanusamic acid has been postulated as an indicator of glucosinolate breakdown in intact tissue [51]. It can result from metabolism of structurally different glucosinolates. Therefore, future experiments should test the presence of indolic amines and raphanusamic acid in root homogenates of *nsp1-1* in comparison to the wild-type. It would also be interesting to analyze these metabolites in root extracts prepared under conditions that prevent glucosinolate hydrolysis in order to establish glucosinolate breakdown pathways in intact tissue. Furthermore, reactions of the indolic isothiocyanates and carbinols with other nucleophiles, e.g., ascorbate, should be considered in future experiments [19,22,28].

In conclusion, we present a GC-MS-based method for sensitive detection and quantification of the carbinols and nitriles derived from 1MOI3M, I3M and 4MOI3M in plant extracts. Future experiments might extend this study to various glucosinolate-containing crop plants in order to evaluate the formation of the various breakdown products of indole glucosinolates upon homogenization, other steps of processing or ingestion. The method might also be helpful for product quantification in experiments aimed at biochemical characterization of purified enzymes involved in glucosinolate breakdown in vitro. Applying this method to *A. thaliana*, we demonstrated that carbinols are more dominant among the detected products in rosette homogenates than in root homogenates. Indolic nitrile formation in rosette homogenates is entirely due to NSP1. In root homogenates, NSP1 seems to be the major NSP responsible for indolic nitrile formation, with no contribution from NSP3. It remains to be evaluated whether indolic nitriles detected in homogenates of *nsp1-1* roots are due to the activity of NSP4. Deficiency of indolic nitrile formation in roots is likely associated with the formation of products not detected by our method, especially in case of I3M and 4MOI3M. Therefore, future experiments should be directed at profiling indolic metabolites of these glucosinolates in *nsp1-1* vs. wild-type roots and root homogenates. Establishment of a method for quantification of nitriles and carbinols derived from 1MOI3M, I3M and 4MOI3M is a necessary first step to assess the formation and biological roles of these products in plants.

## Figures and Tables

**Table 1:** Substitution patterns and abbreviations of indole glucosinolates, as well as the derived carbinols and nitriles. The glucosinolates, carbinols and nitriles considered in this study are listed. The core structures are shown in Figure 1.

R^1^	R^2^	Glucosinolate	Carbinol	Nitrile
H	H	I3M: indol-3-ylmethyl glucosinolate (glucobrassicin)	**1**, I3C: indole-3-carbinol	**2**, I3ACN: indole-3-acetonitrile
OCH_3_	H	1MOI3M: 1-methoxyindol-3-ylmethyl glucosinolate (neoglucobrassicin)	**3**, 1MOI3C: 1-methoxyindole-3-carbinol	**4**, 1MOI3ACN: 1-methoxyindole-3-acetonitrile
H	OCH_3_	4MOI3M: 4-methoxyindol-3-ylmethyl glucosinolate	**5**, 4MOI3C: 4-methoxyindole-3-carbinol	**6**, 4MOI3ACN: 4-methoxyindole-3-acetonitrile

## Data Availability

Not applicable.

## Supplementary Materials

The following supporting information can be downloaded at: https://www.mdpi.com/article/10.3390/molecules27228042/s1. Supplementary Schemes; Supplementary Materials Scheme S1: Synthesis of 1MOI3C based on [41,42]. Suppl. Scheme S2: Synthesis of 1MOI3ACN based on [41,43]. Suppl. Scheme S3: Synthesis of 4MOI3C based on [42]. Suppl. Scheme S4: Synthesis of 4MOI3ACN based on [43]. Supplementary Data: Suppl. Data S1: ESI-MS and NMR spectra of synthesized standards; Suppl. Data S2: GC-EI-MS spectra of standards after complete silylation. Supplementary Figures: Suppl. Figure S1: Evaluation of different incubation times and temperatures for the derivatization of indolic breakdown product standards. Suppl. Figure S2: Indole glucosinolate content in *A. thaliana* wild-type, *nsp1-1* and *nsp3-2* roots and amount of detected indolic breakdown products in root homogenates. Suppl. Figure S3: Indole glucosinolate content in *A. thaliana* wild-type, *nsp1-1* and *nsp3-2* rosettes and amount of detected indolic breakdown products in rosette homogenates. Suppl. Figure S4: *A. thaliana* root and rosette homogenates contain traces of homomeric and heteromeric di(indol-3-yl)methane derivatives. Suppl. Figure S5: Presence of putative carbinol methyl ethers in derivatized extracts of root homogenates from *A. thaliana* Col-0. *Supplementary Tables*: Suppl. Table S1: HPLC gradient for evaluation of purity of synthesized standards. Suppl. Table S2: Evaluation of Response Factors (RFs) for derivatized indole glucosinolate breakdown product standards. Suppl. Table S3: Homomeric and heteromeric di(indol-3-yl)methane derivatives detected by GC-MS in a mixture generated from synthesized carbinols. Suppl. Table S4: HPLC gradient 1 for analysis of desulfoglucosinolates. Suppl. Table S5: HPLC gradient 2 for analysis of desulfoglucosinolates. Suppl. Table S6: Presence of traces of di(indol-3-yl)methane derivatives in plant homogenates.
