# Supplementary material for: The Impact of Nitrile-Specifier Proteins on Indolic Carbinol and Nitrile Formation in Homogenates of Arabidopsis thaliana
Source: Molecules. 2022 Nov 19;27(22):8042. doi: 10.3390/molecules27228042 (PMC9696369; doi:10.3390/molecules27228042)
Supplement: Supplementary file 1 [file molecules-27-08042-s001.zip › molecules-1982285-supplementary.pdf]

The impact of nitrile-specifier proteins on indolic carbinol and nitrile formation in homogenates of *Arabidopsis thaliana*

Eleanor C. M. Chroston, Annika Hielscher, Matthias Strieker, Ute Wittstock

Supplementary Materials

Supplementary Schemes

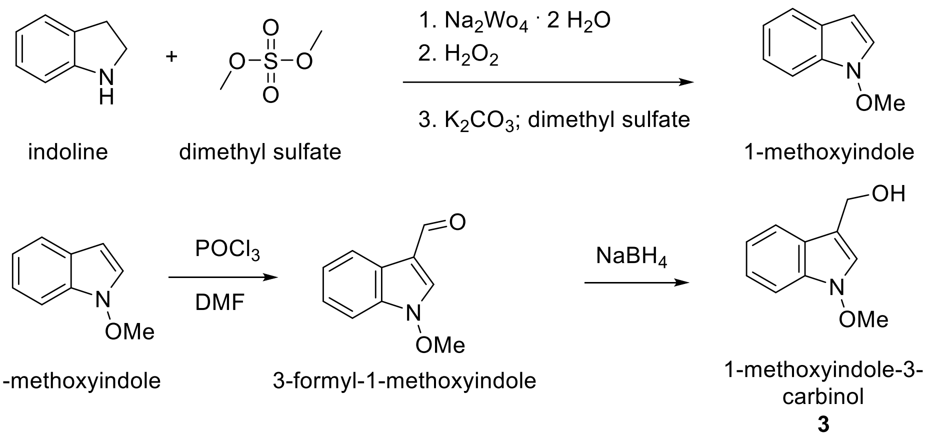

Suppl. Scheme S1: Synthesis of 1MOI3C based on [41, 42].

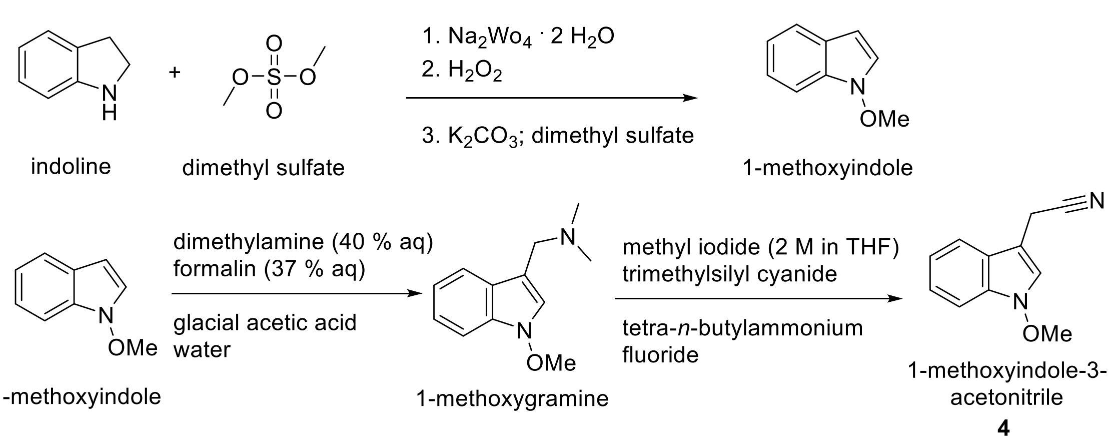

Suppl. Scheme S2: Synthesis of 1MOI3ACN based on [41, 43].

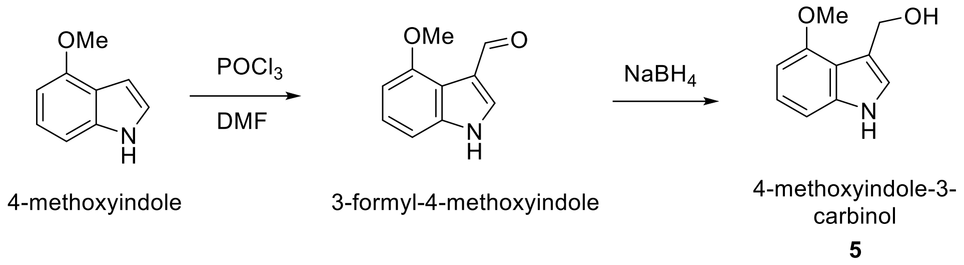

Suppl. Scheme S3: Synthesis of 4MOI3C based on [42].

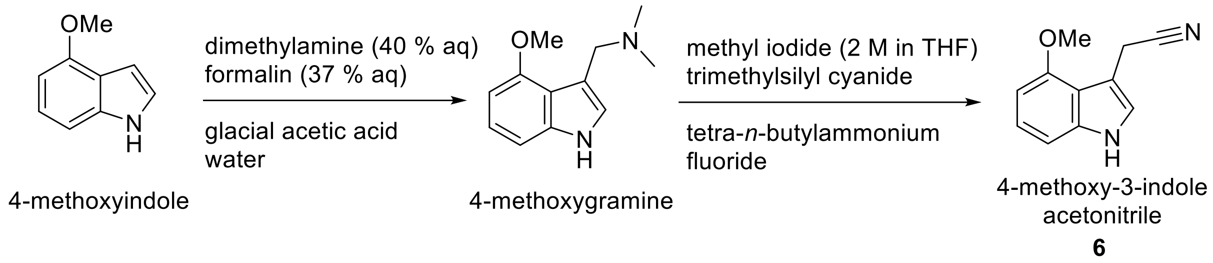

Suppl. Scheme S4: Synthesis of 4MOI3ACN based on [43].

Supplementary Data

Suppl. Data S1: ESI-MS and NMR spectra of synthesized standards

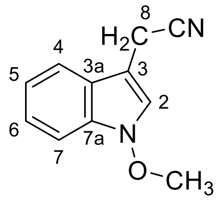

1-Methoxyindole-3-acetonitrile, orange oil. ^{1}H NMR (500 MHz, $CDCl_3$, δ, ppm): 7.56 (1H, dt, *J* = 7.9, 0.9 Hz, H-4), 7.45 (1H, dt, *J* = 8.2, 0.9 Hz, H-7), 7.31 (1H, t, *J* = 0.9 Hz, H-2), 7.30 (1H, ddd, *J* = 8.3, 7.0, 1.0 Hz, H-6), 7.18 (1H, ddd, *J* = 8.1, 7.0, 0.9 Hz, H-5), 4.09 (3H, s, -OCH_3), 3.81 (2H, d, *J* = 1.2 Hz, H-8). ^{13}C NMR (125 MHz, $CDCl_3$, δ, ppm): 132.3 (C, C-7a), 123.3 (CH, C-2), 122.4 (C, C-3a), 121.7 (CH, C-6), 120.4 (CH, C-5), 118.3 (CH, C-4), 117.8 (C, CN), 108.7 (CH, C-7), 100.3 (C, C-3), 66.1 (CH_3, OCH_3), 14.3 (CH_2, C-8). ESI-MS: *m/z* 187.1 $[M+H]^+$.

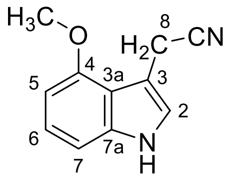

4-Methoxyindole-3-acetonitrile, brown solid. ^{1}H NMR (500 MHz, $CDCl_3$, δ, ppm): 8.11 (1H, s broad, NH), 7.12 (1H, t, *J* = 8.0 Hz, H-6), 7.08 (1H, m, H-2), 6.96 (1H, d, *J* = 8,2 Hz, H-7), 6.50 (1H, d, *J* = 7.8 Hz, H-5), 3.91 (3H, s, -OCH_3), 4.05 (2H, d, *J* = 1.2 Hz, H-8). ^{13}C NMR (125 MHz, $CDCl_3$, δ, ppm): 154.4 (C, C-4), 137.8 (C, C-7a), 123.7 (CH, C-6), 121.3 (CH, C-2), 119.1 (C, C-3a), 116.3 (C, CN), 105.3 (C, C-3), 104.6 (CH, C-7), 99.9 (CH, C-5), 55.2 (CH_3, OCH_3), 16.0 (CH_2, C-8). ESI-MS: *m/z* 187.1 $[M+H]^+$, 209.0 $[M+Na]^+$.

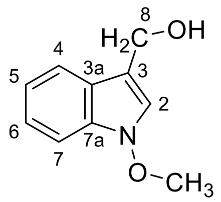

1-Methoxyindole-3-methanol, beige oil. ^{1}H NMR (400 MHz, CD_3OD, δ, ppm): 7.65 (1H, dt, *J* = 8.0, 0.8 Hz, H-4), 7.39 (1H, dt, *J* = 8.3, 0.8 Hz, H-7), 7.37 (1H, s, H-2), 7.20 (1H, ddd, *J* = 8.3, 7.0, 1.0 Hz, H-6), 7.07 (1H, ddd, *J* = 8.1, 7.0, 1.0 Hz, H-5), 4.75 (2H, d, *J* = 0.6 Hz, H-8), 4.05

(3H, s, -OCH_3). ^{13}C NMR (100 MHz, CD_3OD, δ, ppm): 134.0 (C, C-7a), 124.6 (C, C-3a), 123.5 (CH, C-2), 123.1 (CH, C-6), 120.9 (CH, C-5), 120.3 (CH, C-4), 113.2 (C, C-3), 109.2 (CH, C-7), 66.2 (CH_3, OCH_3), 56.9 (CH_2, C-8). ESI-MS: *m/z* 200.0 $[M+Na]^+$.

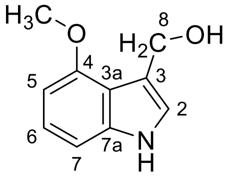

4-Methoxyindole-3-methanol, pale yellow solid. ^1H NMR (400 MHz, CD_3OD, δ, ppm): 7.06 (1H, s, H-2), 7.01 (1H, t, *J* = 8.0 Hz, H-6), 6.95 (1H, dd, *J* = 8.2, 1.0 Hz, H-7), 6.49 (1H, dd, *J* = 7.6, 0.7 Hz, H-5), 4.85 (2H, d, *J* = 0.6 Hz, H-8), 3.91 (3H, s, -OCH_3). ^{13}C NMR (100 MHz, $CDCl_3$, δ, ppm): 155.4 (C, C-4), 140.0 (C, C-7a), 123.5 (CH, C-6), 122.9 (CH, C-2), 117.9 (C, C-3a), 116.6 (C, C-3), 106.0 (CH, C-7), 100.2 (CH, C-5), 58.7(CH_2, C-8), 55.6 (CH_3, OCH_3). ESI-MS: *m/z* 199.9 $[M+Na]^+$, 214.0 $[M+K]^+$.

Suppl. Data S2: GC-EI-MS spectra of standards after complete silylation. The trimethylsilyl group replacing a reactive hydrogen is indicated in red. IST, internal standard.

I3C (**1**)

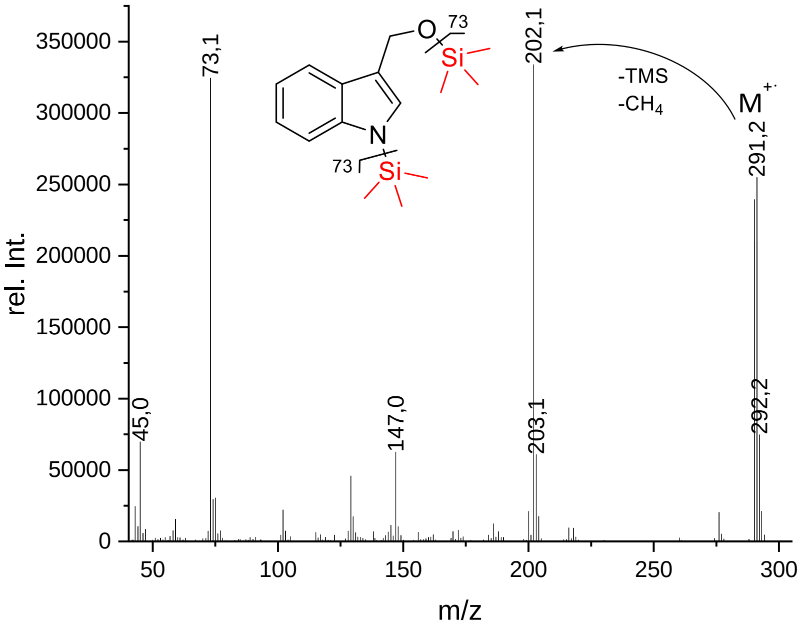

I3ACN (**2**)

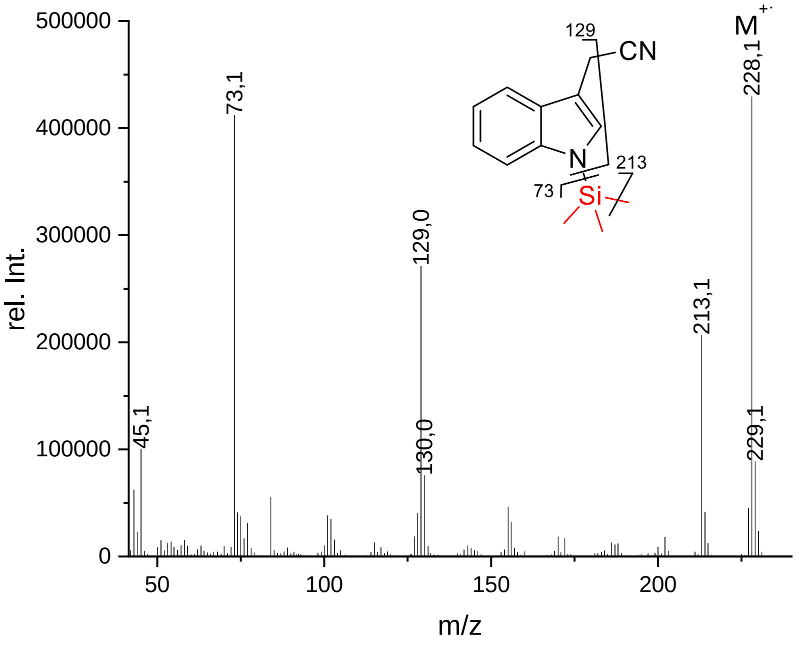

1MOI3C (**3**)

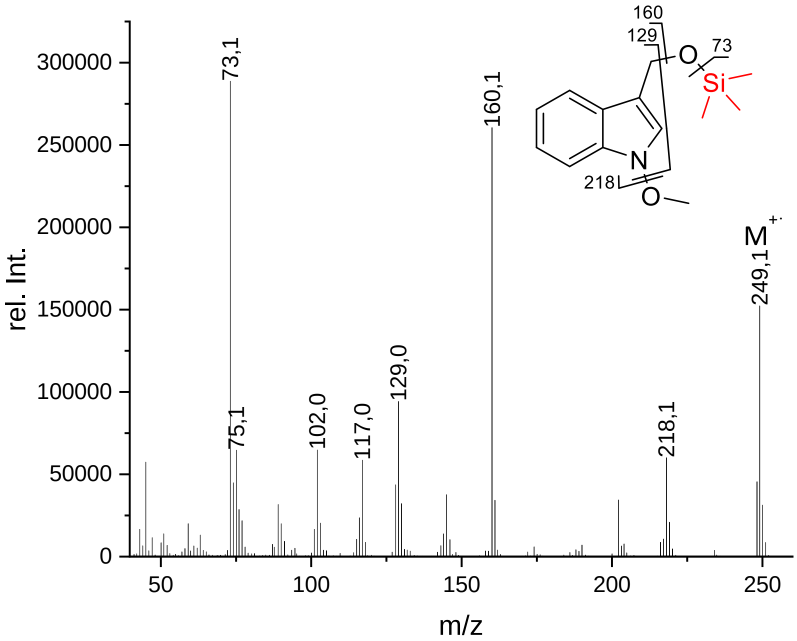

1MOI3ACN (**4**)

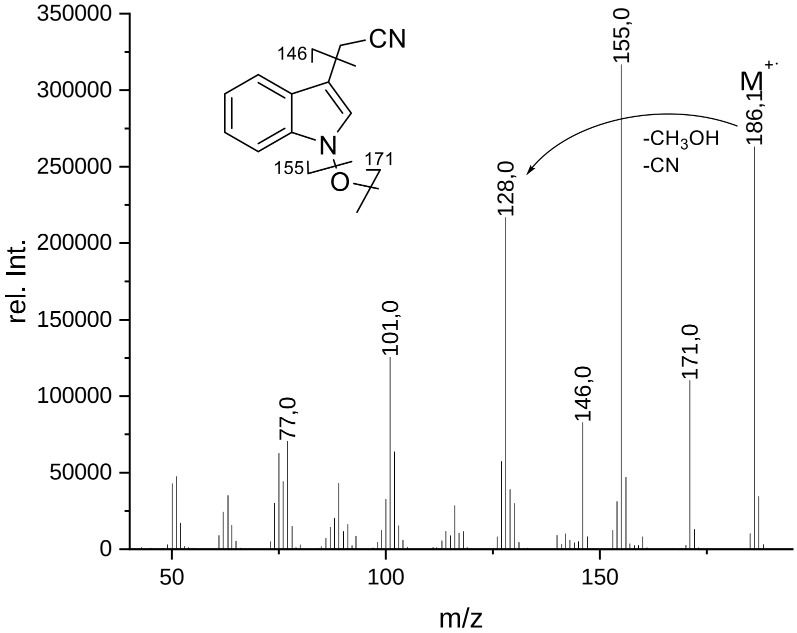

4MOI3C (**5**)

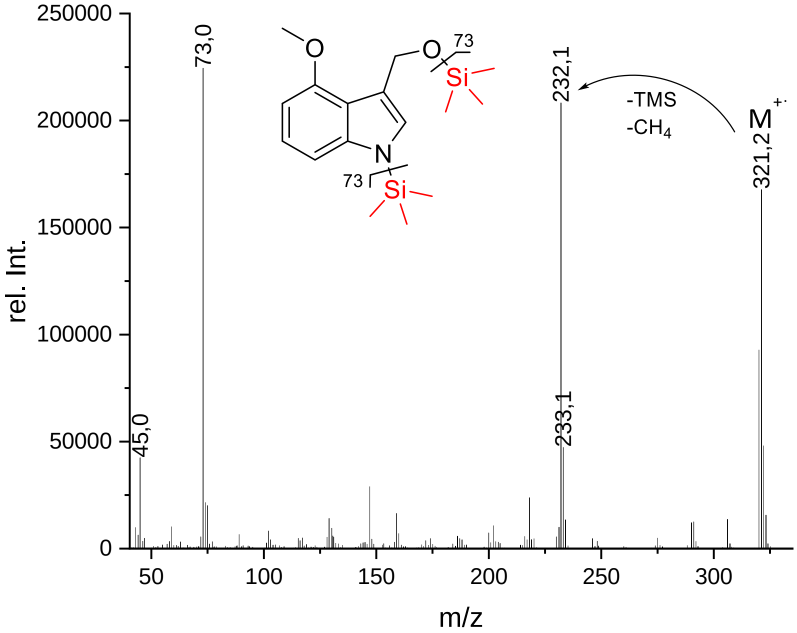

4MOI3ACN (**6**)

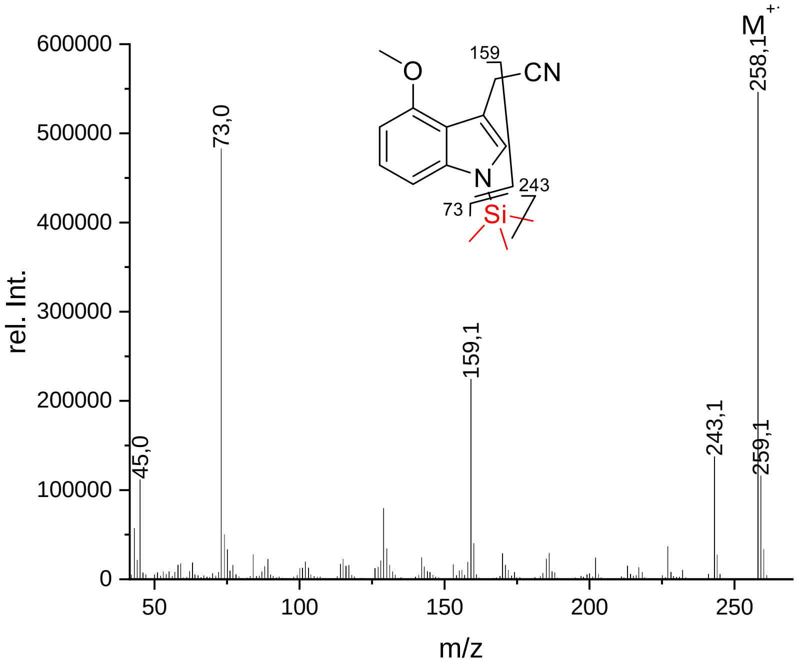

I3CN (IST)

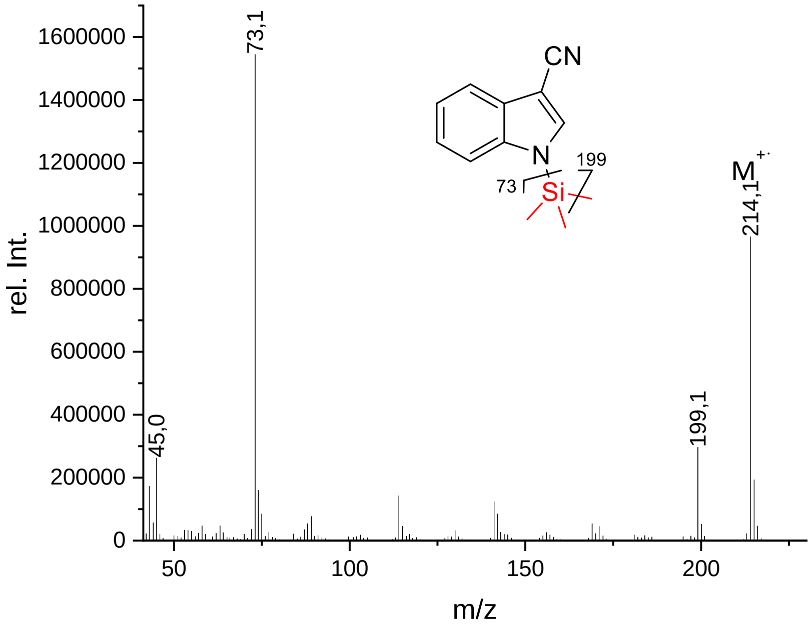

73,1
CN
N
199
73
Si
M+·
214,1
199,1
45,0
1600000
1400000
1200000
1000000
800000
600000
400000
200000
0
rel. Int.
50
100
150
200
m/z

Supplementary Figures

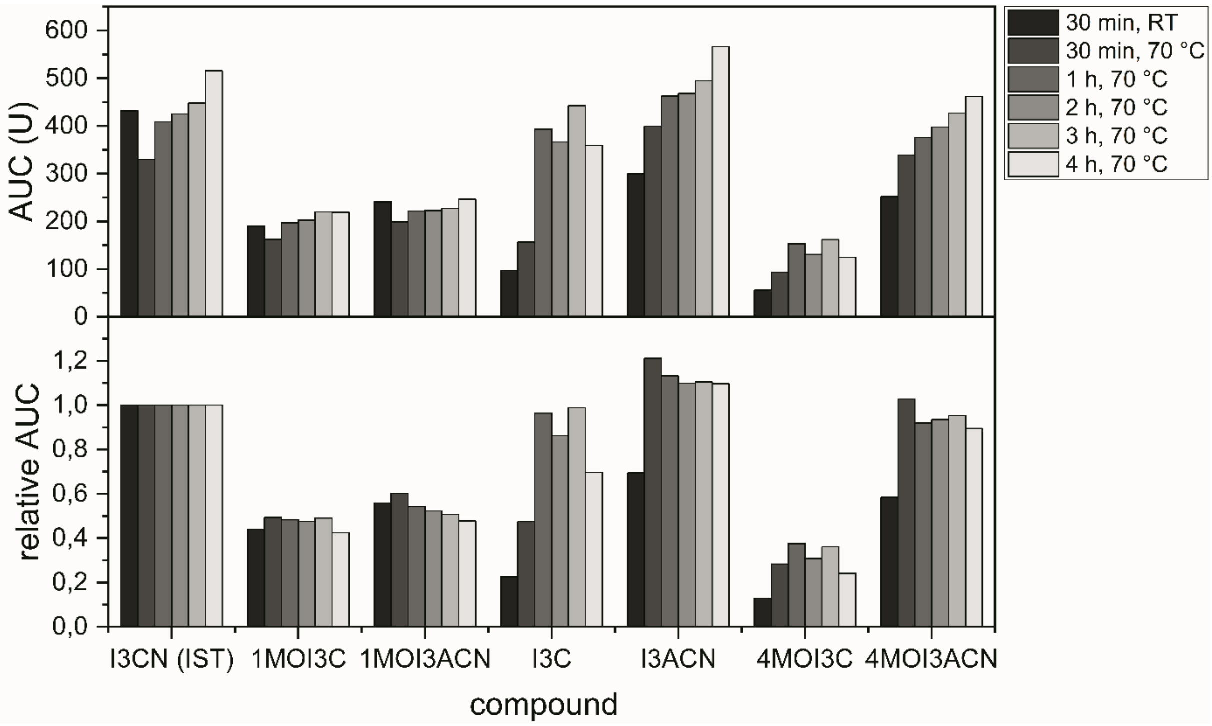

Suppl. Figure S1: Evaluation of different incubation times and temperatures for the derivatization of indolic breakdown product standards. Mixtures of 50 nmol of each standard compound were incubated with 75 µl MSTFA/pyridine (2+1) for varying amounts of time at room temperature (RT) or 70 °C and directly analyzed by GC-FID. Shown are absolute peak areas (top) and peak areas relative to I3CN (bottom).

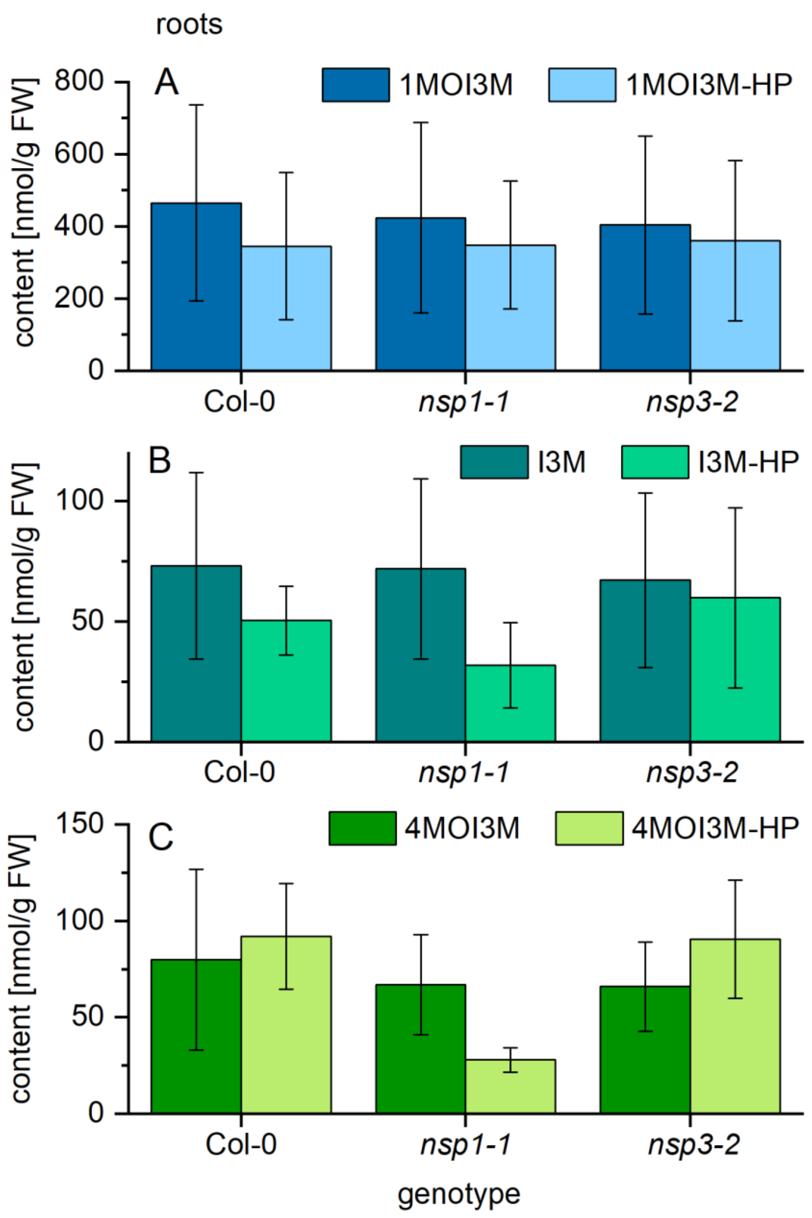

Suppl. Figure S2: Indole glucosinolate content in *A. thaliana* wild-type, *nsp1-1* and *nsp3-2* roots and amount of detected indolic breakdown products in root homogenates. Separate sets of plants were used for determination of glucosinolate content by HPLC of desulfoglucosinolates (**A**: 1MOI3M; **B**: I3M; **C**: 4MOI3M) and for quantification of corresponding indolic carbinols and nitriles (**A**: 1MOI3M-HP; **B**: I3M-HP; **C**: 4MOI3M-HP) by GC-MS after derivatization. Material from six plants was pooled for one sample per experiment. Shown are means ± SD of *N*=4 independent experiments (glucosinolates) and *N*=6 independent experiments (hydrolysis products).

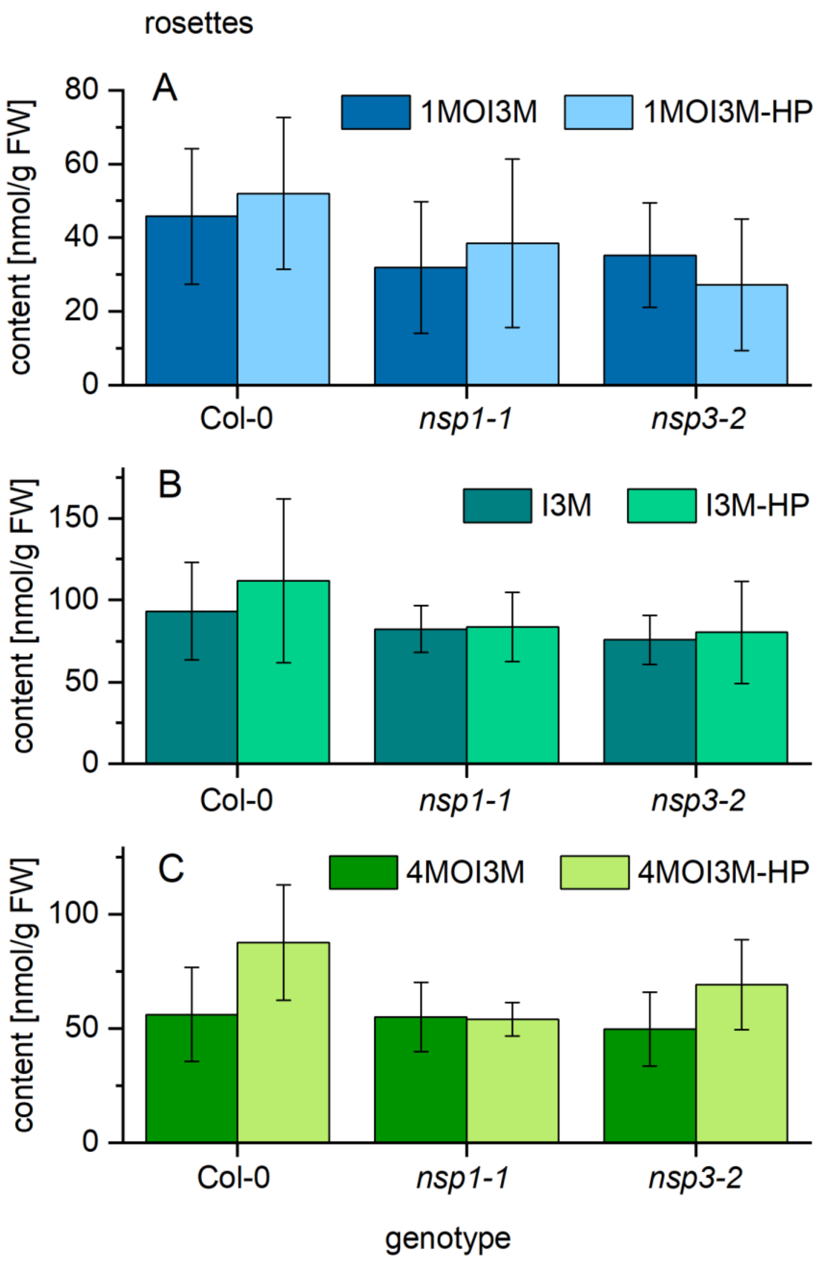

Suppl. Figure S3: Indole glucosinolate content in *A. thaliana* wild-type, *nsp1-1* and *nsp3-2* rosettes and amount of detected indolic breakdown products in rosette homogenates. Separate sets of plants were used for determination of glucosinolate content by HPLC of desulfoglucosinolates (**A**: 1MOI3M; **B**: I3M; **C**: 4MOI3M) and for quantification of corresponding indolic carbinols and nitriles (**A**: 1MOI3M-HP; **B**: I3M-HP; **C**: 4MOI3M-HP) by GC-MS after derivatization. Rosettes of three plants were pooled for one sample for glucosinolate quantification, shown are means ± SD of *N*=4 independent experiments. Hydrolysis products were quantified in homogenates of individual rosettes in three independent experiments with three replicates each (*N*=9), shown are means ± SD.

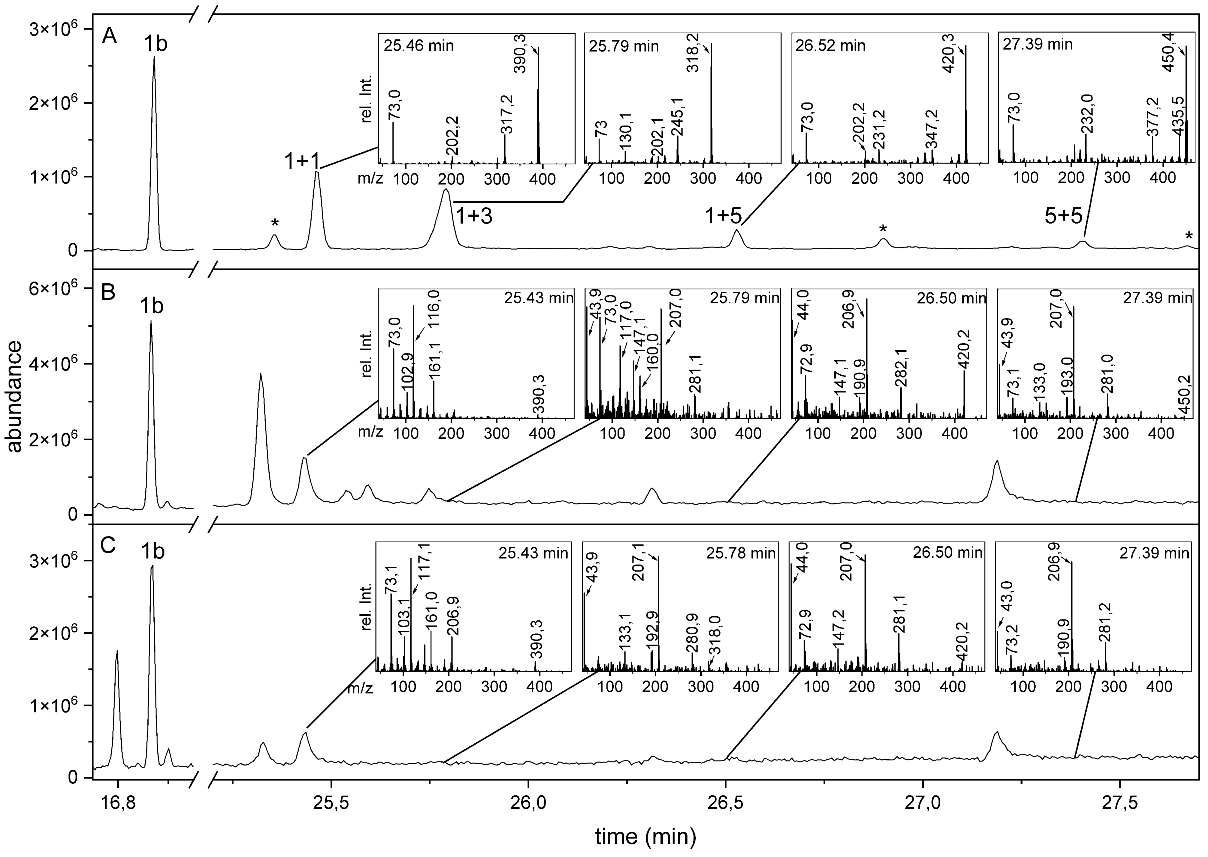

Suppl. Fig. S4: *A. thaliana* root and rosette homogenates contain traces of homomeric and heteromeric di(indol-3-yl)methane derivatives. Di(indol-3-yl)methane derivatives (compound abbreviations as in Suppl. Table S3) were generated from a mixture of I3C, 1MOI3C, and 4MOI3C that was left at pH 5 for 17 h, followed by derivatization (see Materials and Methods) and GC-MS analysis (**A**) for comparison with *A. thaliana* rosette (**B**) and root (**C**) homogenates. One representative chromatogram is shown for each organ. Mass spectra of the peaks representing di(indol-3-yl)methane derivatives are depicted in **A** for comparison with those detected at the same retention times in **B** and **C**. Sample **A** was generated in 2022 while chromatograms in **B** and **C** were recorded in 2021. For reference, the peak of **1b** (fully silylated I3C) is included in the chromatograms. Asteriscs indicate peaks in **A** with mass spectra lacking signals for di(indol-3-yl)methane derivatives.

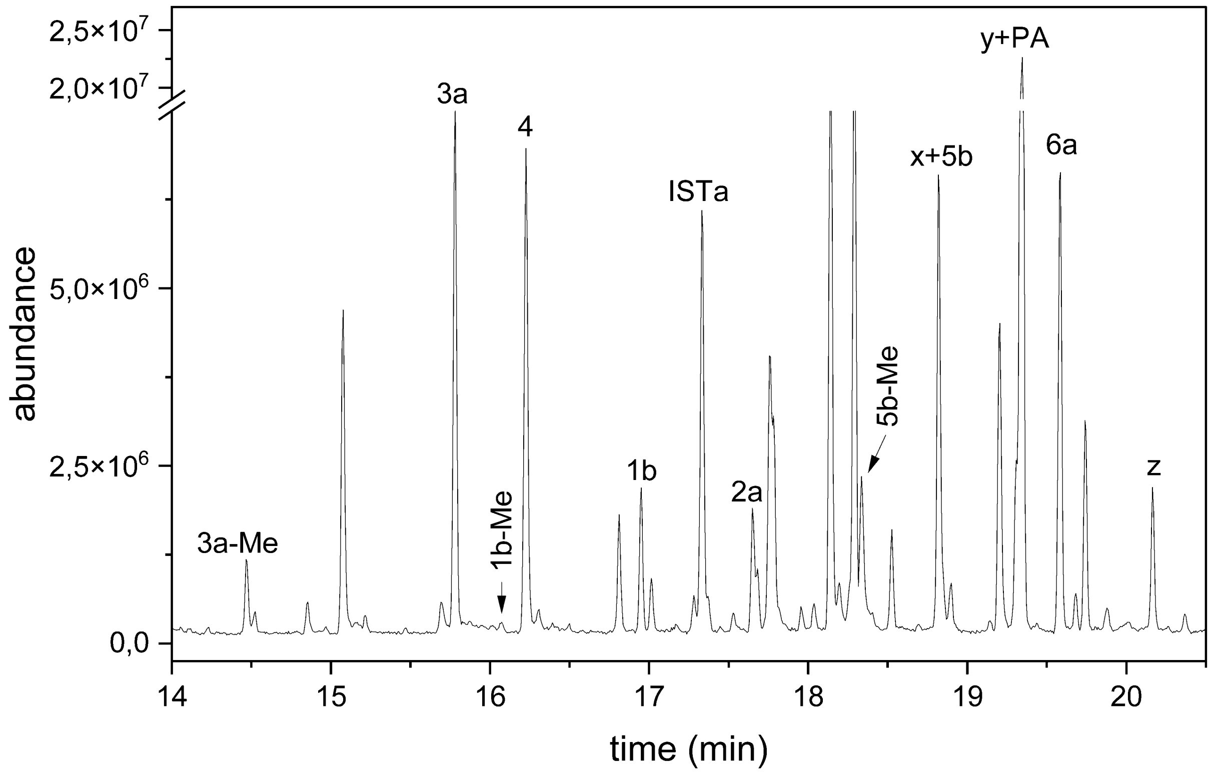

Suppl. Figure S5: Presence of putative carbinol methyl ethers in derivatized extracts of root homogenates from *A. thaliana* Col-0. Root homogenates were extracted with dichloromethane. The extracts were subjected to derivatization and analyzed by GC-MS. Shown is a TIC chromatogram. Numbers refer to compounds in Table 1; IST, Internal Standard I3CN, x, unidentified compound. -Me indicates the methyl ether of the respective compound. Peak y represents threefold silylated 4OHI3C plus silylated palmitic acid (PA), peak z represents twofold silylated 4OHI3ACN.

Supplementary Tables

Suppl. Table S1: HPLC gradient for evaluation of purity of synthesized standards (solvent A: water, solvent B: acetonitrile).

time [min]	% (v/v) B
0-5	0-100
5-6	100

Suppl. Table S2: Evaluation of Response Factors (RFs) for derivatized indole glucosinolate breakdown product standards. RIC-peak areas of the molecular ions were plotted against the molar amounts. Data were subjected to linear regression, the coefficients of determination are given. In experiment 1 (MR1), measurements were done with four dilution series (D1-D4), and data were pooled to generate one regression line per compound. In experiment 2 (MR2), three dilution series (D1-D3) were analyzed with three replicates each; data for replicates were pooled to obtain one regression line per dilution series and compound. The slope of the regression line was used to calculate the RF as slope ratio (internal standard/analyte).

**coefficient of determination (R^2)**						
**compound**	**1**	**2**	**3**	**4**	**5**	**6**
R^2 MR1 D1-D4	0,98	0,99	0,94	0,93	0,97	0,97
R^2 MR2 D1	0,98	0,99	0,99	0,98	0,99	0,98
R^2 MR2 D2	1,00	0,99	0,99	1,00	1,00	0,98
R^2 MR2 D3	1,00	0,98	1,00	1,00	1,00	0,98
**response factor (RF)**						
**compound**	**1**	**2**	**3**	**4**	**5**	**6**
RF MR1 D1-D4	0,88	0,83	4,01	3,13	0,99	0,73
RF MR2 D1	0,90	0,95	1,86	1,60	0,94	0,96
RF MR2 D2	1,15	0,90	2,25	1,95	1,19	0,88
RF MR2 D3	1,13	0,93	2,11	1,79	1,18	0,88
**mean RF**	**1,01**	**0,90**	**2,56**	**2,12**	**1,08**	**0,86**
SE of mean	0,07	0,03	0,49	0,35	0,06	0,05

Suppl. Table S3. Homomeric and heteromeric di(indol-3-yl)methane derivatives detected by GC-MS in a mixture generated from synthesized carbinols. Carbinols were left at pH 5 for 17 h, followed by derivatization (see Materials and Methods). Instead of chemical names, the carbinols are listed from which the di(indol-3-yl)methane (DIM) derivatives are derived, together with an abbreviation (abbrev.). For detected compounds, *m/z* of the corresponding base peak and retention times (t_R) are listed. Parentheses indicate that the base peak was not found, the calculated value is then given in parentheses followed by the *m/z* of a characteristic fragment ion, if fragments were detected.

carbinol educts	DIM abbrev.	TMS groups	t_R [min]	*m/z*	remarks
I3C + I3C	**1+1**	2	25.46	390	
1MOI3C + 1 MOI3C	**3+3**	0	n.a.	(306)	not detected
4MOI3C + 4MOI3C	**5+5**	2	27.39	450	
I3C + 1MOI3C	**1+3**	1	25.79	(348) 318	
I3C + 4MOI3C	**1+5**	2	26.52	420	
1MOI3C + 4MOI3C	**3+5**	1	n.a.	(378)	not detected

Suppl. Table S4: HPLC gradient 1 for analysis of desulfoglucosinolates (solvent A: water, solvent B: acetonitrile).

time [min]	% (v/v) B
0-1	1.5
1-5	1.5-5
5-7	5-7
7-17	7-21
17-22	21-29
22-29	29-43
29-34	43-93
34-39	93

Suppl. Table S5: HPLC gradient 2 for analysis of desulfoglucosinolates (solvent A: water, solvent B: methanol).

time [min]	% (v/v) B
0-10	5
10-70	5-60
70-71	60-100
71-73.5	100

Suppl. Table S6: Presence of traces of di(indol-3-yl)methane derivatives in plant homogenates. Plant homogenates were prepared, derivatized and analyzed by GC-MS as described in Materials and Methods. Chromatograms were inspected for di(indol-3-yl)methane derivatives based on the retention times and *m/z* determined in a standard mixture (Suppl. Table S3). Compound abbreviations are given in Suppl. Table S3. Compounds in brackets were not detected in the standard mixture (Suppl. Table S3), therefore chromatograms were inspected for the expected *m/z* regardless of retention time. “--", not detectable.

	**1+1**	[**3+3**]	**5+5**	**1+3**	**1+5**	[**3+5**]
roots Col-0						
sample 1	traces	--	--	--	--	--
sample 2	traces	--	--	--	--	--
sample 3	--	--	--	--	--	--
sample 4	--	--	--	--	--	--
sample 5	traces	--	--	--	--	--
sample 6	--	--	--	--	--	--
roots nsp1-1						
sample 1	--	--	--	--	--	--
sample 2	--	--	--	--	--	--
sample 3	traces	--	--	traces	traces	--
sample 4	--	--	--	--	--	--
sample 5	traces	--	--	traces	--	--
sample 6	--	--	--	--	--	--
roots nsp3-2						
sample 1	--	--	--	--	--	--
sample 2	--	--	--	--	--	--
sample 3	traces	--	--	traces	--	--
sample 4	traces	--	--	--	--	--
sample 5	traces	--	--	--	--	--
sample 6	--	--	--	--	--	--
rosettes Col-0						
sample 1	traces	--	traces	--	traces	--
sample 2	traces	--	--	--	--	--
sample 3	traces	--	--	--	traces	--
sample 4	traces	--	traces	--	traces	--
sample 5	traces	--	traces	--	traces	--
sample 6	traces	--	--	--	--	--
sample 7	traces	--	--	--	--	--

sample 8	--	--	--	--	--	--
sample 9	--	--	--	--	--	--
rosettes nsp1-1						
sample 1	traces	--	traces	traces	traces	--
sample 2	--	--	--	--	--	--
sample 3	--	--	--	--	traces	--
sample 4	traces	--	traces	--	traces	--
sample 5	traces	--	traces	--	traces	--
sample 6	traces	--	traces	--	traces	--
sample 7	traces	--	--	--	traces	--
sample 8	traces	--	traces	--	traces	--
sample 9	--	--	--	--	--	--
rosettes nsp3-2						
sample 1	--	--	--	--	--	--
sample 2	traces	--	--	--	--	--
sample 3	traces	--	--	--	--	--
sample 4	traces	--	traces	--	traces	--
sample 5	traces	--	traces	--	traces	--
sample 6	traces	--	traces	--	traces	--
sample 7	traces	--	--	--	traces	--
sample 8	traces	--	--	--	--	--
sample 9	--	--	--	--	--	--
